# Protective Effect of Sulforaphane on Oxidative Stress and Mitochondrial Dysfunction Associated with Status Epilepticus in Immature Rats

**DOI:** 10.1007/s12035-022-03201-x

**Published:** 2023-01-04

**Authors:** Jaroslava Folbergrová, Pavel Ješina, Jakub Otáhal

**Affiliations:** Institute of Physiology of the Czech Academy of Sciences, Vídeňská 1083, 142 20, Prague 4, Czech Republic

**Keywords:** Immature rats, Status epilepticus, Oxidative stress, Mitochondrial dysfunction, Sulforaphane, Protection

## Abstract

The present study aimed to elucidate the effect of sulforaphane (a natural isothiocyanate) on oxidative stress and mitochondrial dysfunction during and at selected periods following status epilepticus (SE) induced in immature 12-day-old rats by Li-pilocarpine. Dihydroethidium was employed for the detection of superoxide anions, immunoblot analyses for 3-nitrotyrosine (3-NT) and 4-hydroxynonenal (4-HNE) levels and respiratory chain complex I activity for evaluation of mitochondrial function. Sulforaphane was given i.p. in two doses (5 mg/kg each), at PD 10 and PD 11, respectively. The findings of the present study indicate that both the acute phase of SE and the early period of epileptogenesis (1 week and 3 weeks following SE induction) are associated with oxidative stress (documented by the enhanced superoxide anion production and the increased levels of 3-NT and 4-HNE) and the persisting deficiency of complex I activity. Pretreatment with sulforaphane either completely prevented or significantly reduced markers of both oxidative stress and mitochondrial dysfunction. Since sulforaphane had no direct anti-seizure effect, the findings suggest that the ability of sulforaphane to activate Nrf2 is most likely responsible for the observed protective effect. Nrf2-ARE signaling pathway can be considered a promising target for novel therapies of epilepsy, particularly when new compounds, possessing inhibitory activity against protein–protein interaction between Nrf2 and its repressor protein Keap1, with less “off-target” effects and, importantly, with an optimal permeability and bioavailability properties, become available commercially.

## Introduction

Epilepsy is one of the most common neurological disorders of the brain, characterized by spontaneous recurrent seizures. It is particularly frequent in infants and children where epileptic activity may lead to adverse effects on brain maturation and serious functional consequences later in life, such as various cognitive defects [1, 2].

Current therapies are mostly symptomatic and not fully effective. In addition, a large percentage of epileptic patients are refractory to available antiseizure drugs (ASD) [3]. Hence, there is a need to develop more effective therapies. This requires a better understanding of the key mechanisms underlying the disease and identifying new potential targets for novel and more efficient therapies.

It is well established that free radicals, oxidative stress, and mitochondrial dysfunction are implicated in the pathogenesis of many neurological diseases, including epilepsy in adult [4–11] as well as immature animals [12–17]. Many efforts have been aimed at developing substances capable of detoxifying reactive oxygen and/or nitrogen species (ROS and RNS) and their damaging effects [18, 19]. Synthetic metalloporphyrin catalytic antioxidants were considered as potential neuroprotective agents [20–22]. Some of these compounds (e.g., SOD mimetics MnTMPYP and Tempol or a peroxynitrite scavenger and decomposition catalyst FeTPPS) have been reported to prevent or substantially attenuate oxidative stress, neuronal damage, and mitochondrial dysfunction associated with status epilepticus (SE) in adult [7, 23] as well as immature animals [13, 15, 17, 24–27].

It can be assumed that antioxidants interacting with multiple targets and/or possessing the ability to increase the endogenous antioxidant defenses of cells can have a better beneficial effect than single-target therapies and might ensure long-term protection [28]. We have demonstrated recently that both oxidative stress and mitochondrial dysfunction associated with SE in immature rats can be prevented or substantially attenuated after treatment with resveratrol (RSV, 3,5,4′-tri-hydroxy-trans-stilbene), a natural polyphenolic compound present in red wine [17, 26]. It has been reported that RSV besides its direct antioxidant effect [28–30] has multiple cellular effects, interfering with several signaling pathways [29, 31]. Recently, it has been shown [32] that RSV activates Nrf2 (nuclear factor erythroid 2-related factor 2) which is an essential transcription factor that plays a crucial role in cellular defense against oxidative stress [33–35].

Under homeostatic (basal) conditions, Nrf2 is mainly regulated by Kelch-like ECH-associated protein 1 (Keap 1). Keap1 binds in the cytoplasm to Nrf2 and directs it to ubiquitination and subsequent proteasomal degradation. In the presence of electrophilic compounds or oxidative stress conditions, Keap1-Nrf2 interaction is disrupted, and Nrf2 translocates into the nucleus, wherein after heterodimerization with small Maf proteins binds to antioxidant response element (ARE) and leads to the expression of numerous genes encoding for proteins and enzymes with antioxidant, anti-inflammatory, and cytoprotective functions [34, 36, 37].

Nrf2, the master regulator of cellular redox homeostasis, thus seems to be a promising therapeutic target. Indeed, activation of the Nrf2/ARE pathway has been reported to provide protection in several animal models of neurodegenerative diseases in adult animals like Alzheimer’s (AD), Parkinson’s (PD), Huntington’s (HD) disease, ischemic stroke, cancer, and epilepsy [36, 38–44]. Increasing evidence supports the role of Nrf2 in mitochondrial bioenergetics and function [45–49].

Regarding epilepsy, Mazzuferi et al. [41] reported that overexpression of Nrf2 (via adeno-associated virus vector) in adult mice with pilocarpine-induced SE provided a marked protective effect. Furthermore, activation of the Nrf2-ARE pathway by sulforaphane (SFN) was able to suppress the progression of amygdala kindling in adult rats, protect the brain from damage, and ameliorate cognitive impairment [42]. Pauletti et al. [43], using a rat model of acquired epilepsy induced by electrical status epilepticus, reported that transient treatment (for 2 weeks during epileptogenesis) with SFN alone or in combination with N-acetylcysteine reduced oxidative stress, decreased neuronal loss, and rescued cognitive deficits. In addition, this treatment significantly delayed the onset of epilepsy, blocked disease progression between 2 and 5 months following SE, and markedly reduced the frequency of spontaneous seizures evaluated at 5 months. Recently, a novel Nrf2 activator, namely, RTA 408 (the compound belonging to the chemical class of cyanoenone triterpenoids), has been employed by Shekh-Ahmad et al. [44]. In kainic acid-induced SE, RTA 408 administered after SE over 3 days inhibited ROS production, increased glutathione and ATP levels, and prevented neuronal death. Importantly, RTA408 had no direct anti-seizure effect, but it dramatically reduced the frequency of late spontaneous seizures for at least 4 months following SE. Interestingly, a recent study by Shekh-Ahmad et al. [50] has revealed that combination therapy with RTA 408 and AEBSH (NOX2 inhibitor) prevented the development of spontaneous seizures following KA-induced SE.

Numerous classes of compounds, particularly constituents of vegetables and spices, have been identified as activators of Nrf2 [51, 52]. Among others, sulforaphane (SFN), a natural isothiocyanate present in cruciferous vegetables, especially broccoli and its young sprouts, has been reported as a potent activator of Nrf2 [53–57]. The protective effect of SFN was demonstrated in several in vivo models of neurodegenerative, inflammatory, age-related diseases, cerebral ischemia, stroke, and other pathological conditions in the CNS [54–59], including epilepsy [42, 43].

There are a few studies concerning the SFN effect in immature animals. Thus, in the neonatal hypoxic-ischemia model (concerning 7-day-old rat pups), SFN pretreatment reduced brain infarct volume through increasing Nrf2 and HO-1 expression [60]. In the piglet model of neonatal hypoxic-ischemia (HI), Wang et al. [61] reported that systemic administration of SFN induced the translocation of Nrf2 to the nucleus, increased expression of an enzyme involved in glutathione synthesis, and protected vulnerable neurons from HI injury. However, data regarding the SFN effect in immature rats with epileptic seizures, have been according to our knowledge, missing.

We have therefore decided to explore the effect of SFN during status epilepticus (SE) induced in immature 12-day-old rats by Li-pilocarpine and to perform rather an extensive study concerning the effect of SFN pretreatment on EEG activity, cerebral blood flow (CBF), glucose metabolism, and energy metabolites (part I) and oxidative stress and mitochondrial dysfunction (part II). Findings of the part I have already been published [62].

The present study aimed to assess the SFN effect on oxidative stress and mitochondrial dysfunction (part II).

Regarding the effect on oxidative stress, (1) to investigate superoxide anion production in various brain structures in situ in pups during the acute phase of SE and in those surviving 1 week and 3 weeks, respectively, following SE by employing the dihydroethidium (Het) method and (2) to assess oxidative damage of mitochondrial proteins by detecting the levels of 3-nitrotyrosine (3-NT) and 4-hydroxynonenal (4-HNE) by immunoblot analyses.

Regarding the effect of SFN on mitochondrial function, to determine the activity of respiratory chain complex I in isolated mitochondria of pups at similar time intervals following SE as those used for evaluating oxidative stress.

## Materials and Methods

### Material

LiCl and pilocarpine were purchased from Sigma-Aldrich (St. Louis, MO, USA), sulforaphane from APExBIO (USA), and dihydroethidium (hydroethidine) from Thermo Fisher Scientific. Mouse monoclonal anti-nitrotyrosine, clone 1A6 (05–233) from Scintila, rabbit polyclonal anti-4-hydroxynonenal from Alpha diagnostic, HNE 11-S. Other reagents and chemicals were mostly from Sigma-Aldrich (St. Louis, MO, USA).

### Animals

Immature 12-day-old male Wistar rats were used for these experiments. Twelve-day-old rats were chosen because of the level of brain maturation which is comparable to the early postnatal period in human infants [63]. The rat pups were removed from their dams 1 h before the experiment. They were kept in plastic observation chambers on an electrically heated pad at 34 °C (i.e., the temperature of the nest). The protocol of experiments (number 50/2017) was approved by the Animal Care and Use Committee of the Institute of Physiology, Czech Academy of Sciences, in agreement with the Animal Protection Law of the Czech Republic, which is fully compatible with the guidelines of the European Community Council directives 2010/63/EU. All efforts were made to minimize animal suffering and to reduce the number of animals used.

### Induction of Status Epilepticus

To induce Li-pilocarpine (Li-Pilo) SE, LiCl was dissolved in redistilled water and administered i.p. to PD11 immature rats (127 mg/kg). After 24 h, pilocarpine, dissolved in redistilled water, was given i.p. (35 mg/kg) to lithium-pretreated pups. Control animals received corresponding volumes of the appropriate vehicles. In all animals (with Li-Pilo alone and those with Li-Pilo + SFN), latency to the onset of the first occurrence of clonic movements of one or both forelimbs, intensity and frequency of clonic seizures were continuously observed during an approximately 2-h period and then the rat pups were returned to their mothers for selected periods of survival.

### Treatment with Sulforaphane

For evaluating the potential protective effect of sulforaphane, SFN was dissolved in dimethyl sulfoxide (DMSO) and then diluted with PBS (final concentration of DMSO ~ 0.5%). Only freshly prepared solutions were used for applications. SFN was given i.p. in two doses (5 mg/kg each). The first application was done 48 h (at PD 10) and the second dose 24 h (at PD 11) before induction of SE. The dose and the schedule for SFN treatment were selected on the basis of literary data and our pilot experiments (see Fig. 3A).

### Superoxide Anion Determination

Production of superoxide anion (O_2_˙^−^) in different brain regions in situ was determined by the hydroethidium (Het) method [64], adopted for immature rats, as described in our previous work [13]. Het was given by i.p. injection (final concentration 10 mg/kg). One hour after the administration of Het, rat pups were deeply anesthetized with 20% (w/v) urethane and transcardially perfused with 0.01 M phosphate-buffered saline (PBS), pH 7.4, followed by a fixative solution (4% (w/v)) paraformaldehyde in 0.1 M phosphate buffer, pH 7.4. The brains, after removal from the skull, were postfixed for 3 h at 4 °C in the same fixative, then cryoprotected in sucrose of increasing concentrations (10, 20, and 30% (w/v), respectively) in 0.1 M phosphate buffer, pH 7.4 and finally frozen in dry ice. Coronal Sections (50 µm) were cut through the brain in a cryostat and mounted onto the gelatinated slides. All procedures were performed under reduced light.

The level of the oxidized products of Het was assessed microscopically by the detection of their fluorescence (> 600 nm). Pictures of the selected regions of interest (hippocampal fields CA1, CA3, and DG, primary somatosensory cortex, and dorsal thalamus) of the same size and orientation were captured (AP − 3.5 to − 4.0 according to Paxinos and Watson [65]), with a cooled camera mounted onto upright microscope (10 × magnification lens). Camera settings remained unchanged throughout the evaluation of the current set of tissue sections of animals from one experimental day, treated with the same solution of Het. The group comprised always at least three saline controls, three animals with Li-Pilo alone, and three with Li-Pilo + SFN. The fluorescence signal was analyzed using Matlab and Image Processing Toolbox (Matlab, Mathworks, USA). The image of the area of interest (see above) was segmented to withdraw blood vessels, cerebral ventricles, and other regions that do not contain nervous tissue. The integral intensity of the segmented image was calculated. For interindividual and intergroup comparisons, data were normalized by values of the control animals of the current set. Results are expressed as a percentage of saline-treated animals.

### Isolation of Mitochondria

Mitochondrial fractions were isolated from cerebral cortices (weighing ~ 250 mg), by the method of Liang et al. [66], as described in our previous works [15, 24, 25]. Freshly isolated mitochondria were used for protein determination. Aliquots of mitochondria frozen in liquid nitrogen and stored at − 70 °C were used for complex I and citrate synthase (CS) activity measurements (that were performed within 1 week) and for 3-NT and 4-HNE determination.

### Mitochondrial Markers of Oxidative Damage

3-Nitrotyrosine (3-NT) and 4-hydroxynonenal (4-HNE) were determined in mitochondrial samples (5 µl) (containing 20 µg of protein) by immunoblot analyses according to Ansari et al. [67], with slight modifications, as described in our previous work [24]. The fluorescence was detected using ODYSSEY infrared imaging system (LI-COR Biosciences), and the signal was quantified using Aida 3.21 Image Analyzer software.

### Enzyme Assays

Activities of mitochondrial respiratory chain complex I and citrate synthase were measured at 30 °C in a total reaction volume of 1 ml using a Shimadzu 1601 spectrophotometer. Duplicate determinations were carried out with each mitochondrial sample. More details of the assay conditions are described in our previous studies [24, 25]. The activity was expressed as nanomole per minute per milligram of protein. To correct for the potential variations in mitochondrial contents in the samples, mitochondrial chain complex I activities were also expressed as a ratio to citrate synthase (data not shown).

### Protein Determination

Mitochondrial protein concentration was estimated by Bradford’s method, using bovine serum albumin as a standard.

### Statistics

The data were evaluated by ANOVA on ranks with the Student–Newman–Keuls post hoc test. The level of statistical significance was set to 5%.

## Results

### Behavior Pattern of Status Epilepticus

In agreement with our previous studies, i.p. administration of pilocarpine to 12-day-old rats pretreated the day before with LiCl elicited SE characterized by generalized clonic seizures, recurring frequently for about 2-h period, after which gradual dampening of the intensity and frequency occurs. A detailed description is given in our previous studies [15, 62]. SFN pretreatment did not change latency, character, duration, or severity of seizures and mortality (see Supplementary Fig. 1. in our recent study [62]).

### Generation of O_2_˙− and Their Influencing by SFN During the Acute Phase of SE and During the Various Periods of Survival Following SE

**Fig. 1:**
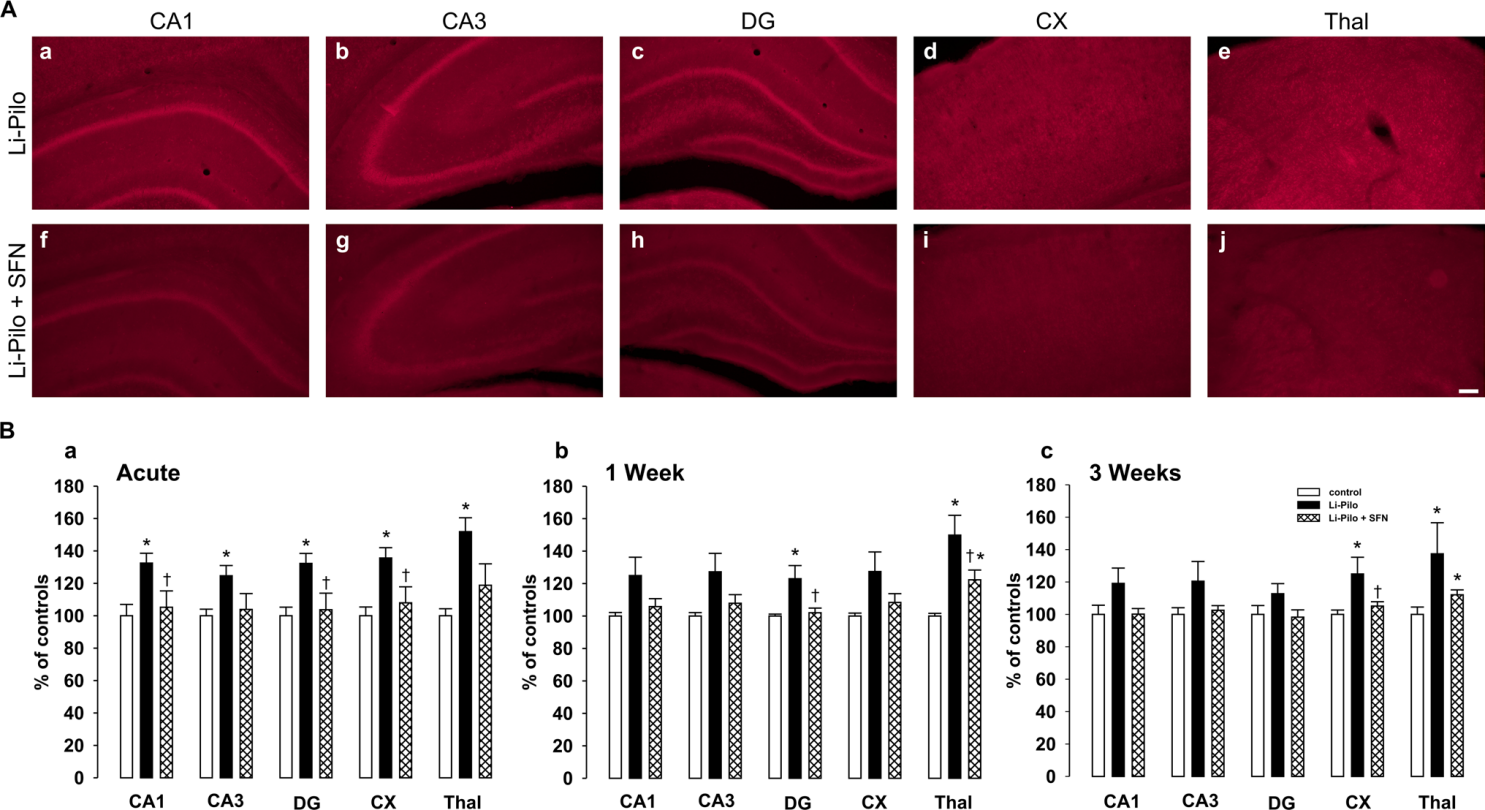
**A** Fluorescence of the oxidized products of hydroethidium (reflecting superoxide anion production), assessed microscopically by fluorescence (> 600 nm), in various brain structures following 60 min lasting SE induced by Li-pilocarpine (Li-Pilo) (acute phase). Upper image: Li-Pilo alone; lower image: Li-Pilo plus SFN. CA1 and CA3, hippocampal fields; DG, dentate gyrus; CX, cerebral cortex; Thal, dorsal thalamus. Scale bar, 100 µm. **B (a)** Effect of sulforaphane (SFN) on superoxide anion formation at 60 min following the onset of SE, induced in immature rats by Li-Pilo (acute phase). White columns, saline-treated controls; black columns, Li-Pilo alone; cross-hatched columns, Li-Pilo plus SFN. Results are expressed in percent, compared to 100% in the control animals. Mean values for 6 animals ± SEM. ^*^P < 0.05 as compared with saline; ^ϯ^*P* < 0.05 as compared with Li-Pilo alone. **(b)** Effect of SFN on superoxide anion formation at 1 week following the induction of SE in immature rats by Li-Pilo. White columns, saline-treated controls; black columns, Li-Pilo alone; cross-hatched columns, Li-Pilo plus SFN. Mean values for 12 animals ± SEM. ^*^P < 0.05 as compared with saline; ^ϯ^*P* < 0.05 as compared with Li-Pilo alone. **(c)** Effect of SFN on superoxide anion formation at 3 weeks following the induction of SE in immature rats by Li-Pilo. White columns, saline-treated controls; black columns, Li-Pilo alone; cross-hatched columns, Li-Pilo plus SFN. Mean values for 6 animals ± SEM. ^*^P < 0.05 as compared with saline; ^ϯ^*P* < 0.05 as compared with Li-Pilo alone

Figure 1A demonstrates an increase in fluorescence of the oxidized products of Het (reflecting superoxide production) in all the studied structures, namely, CA1, CA3, and DG of the hippocampus, cerebral cortex, and thalamus during the acute phase of SE. As can be seen in Fig. 1B (a) (in which the results are expressed in percent compared to 100% in the control animals), the increases are significant in all the studied structures. Pretreatment with SFN resulted in complete prevention of O_2_˙^−^ increase in CA1, DG, and CX, the protection in CA3 and thalamus did not reach the level of statistical significance. Data shown in Fig. 1B (b) and (c) demonstrate the production of O_2_˙^−^ at 1 week and 3 weeks, respectively, following the induction of SE. As can be seen, a significant increase of O_2_˙^−^ formation is evident in DG and Thal at 1-week intervals and, in CX and Thal at 3-week interval, whereas in the remaining structures of both time intervals elevations did not reach the level of statistical significance. The protective effect of SFN on O_2_˙^−^ formation was complete in DG at 1-week interval and in CX at 3-week interval. Protection in the remaining structures did not reach significance.

### Mitochondrial Markers of Oxidative Damage

Two markers of oxidative damage, namely, 3-nitrotyrosine (3-NT) and 4-hydroxynonenal (4-HNE), were measured in isolated mitochondria at selected time points following SE.

**Fig. 2:**
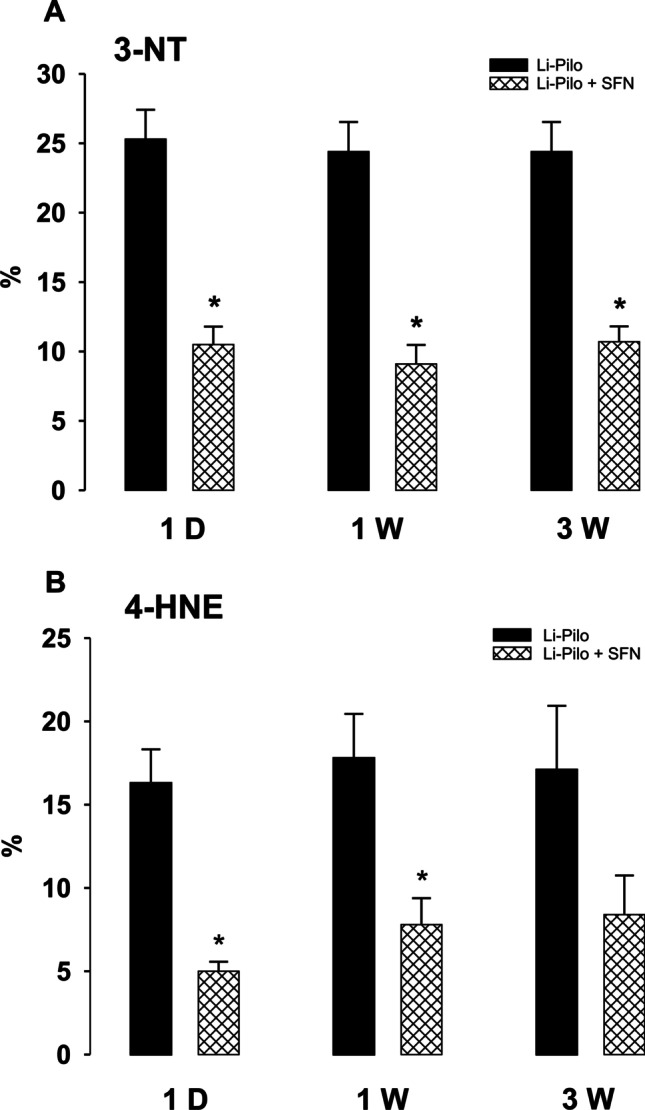
Effect of sulforaphane (SFN) on markers of oxidative damage. **A** 3-Nitrotyrosin (3-NT) in mitochondria isolated from the cerebral cortex of rats at 1 day and 1 and 3 weeks, respectively, following SE induced in immature rats by Li-Pilo. Black columns, Li-Pilo alone; cross-hatched columns, Li-Pilo plus SFN. Results are expressed as increases in percent compared with 100% in the corresponding controls. Mean values for 7 mitochondrial preparations ± SEM. ^*^P < 0.05 as compared with Li-Pilo alone. **B** 4-Hydroxynonenal (4-HNE) in mitochondria isolated from the cerebral cortex of rats at 1 day and 1 and 3 weeks, respectively, following SE induced in immature rats by Li-Pilo. Black columns, Li-Pilo alone; cross-hatched columns, Li-Pilo plus SFN. Results are expressed as increases in percent compared with 100% in the corresponding controls. Mean values for 7 mitochondrial preparations ± SEM. ^*^P < 0.05 as compared with Li-Pilo alone

As illustrated in Fig. 2A, 3-NT levels already increased at 1 day after SE by approximately 24% and the same elevation persisted at 1 week and 3 weeks (the longest period studied), respectively, after SE.

Regarding 4-HNE (Fig. 2B), the increase, corresponding to approximately 17%, can be seen at 1 day and the similar elevation persisted at 1 week and 3 weeks, respectively.

### Effect of SFN on 3-NT and 4-HNE Levels

As demonstrated in Fig. 2A, B, SFN provided a significant attenuation of 3-NT increase in all the studied groups and, concerning 4-HNE, an increase was significantly attenuated by SFN at 1 day and 1 week, at 3 weeks the protection did not reach the significance.

### Mitochondrial Complex I Activities and Their Influencing by SFN During Various Periods Following SE

**Fig. 3:**
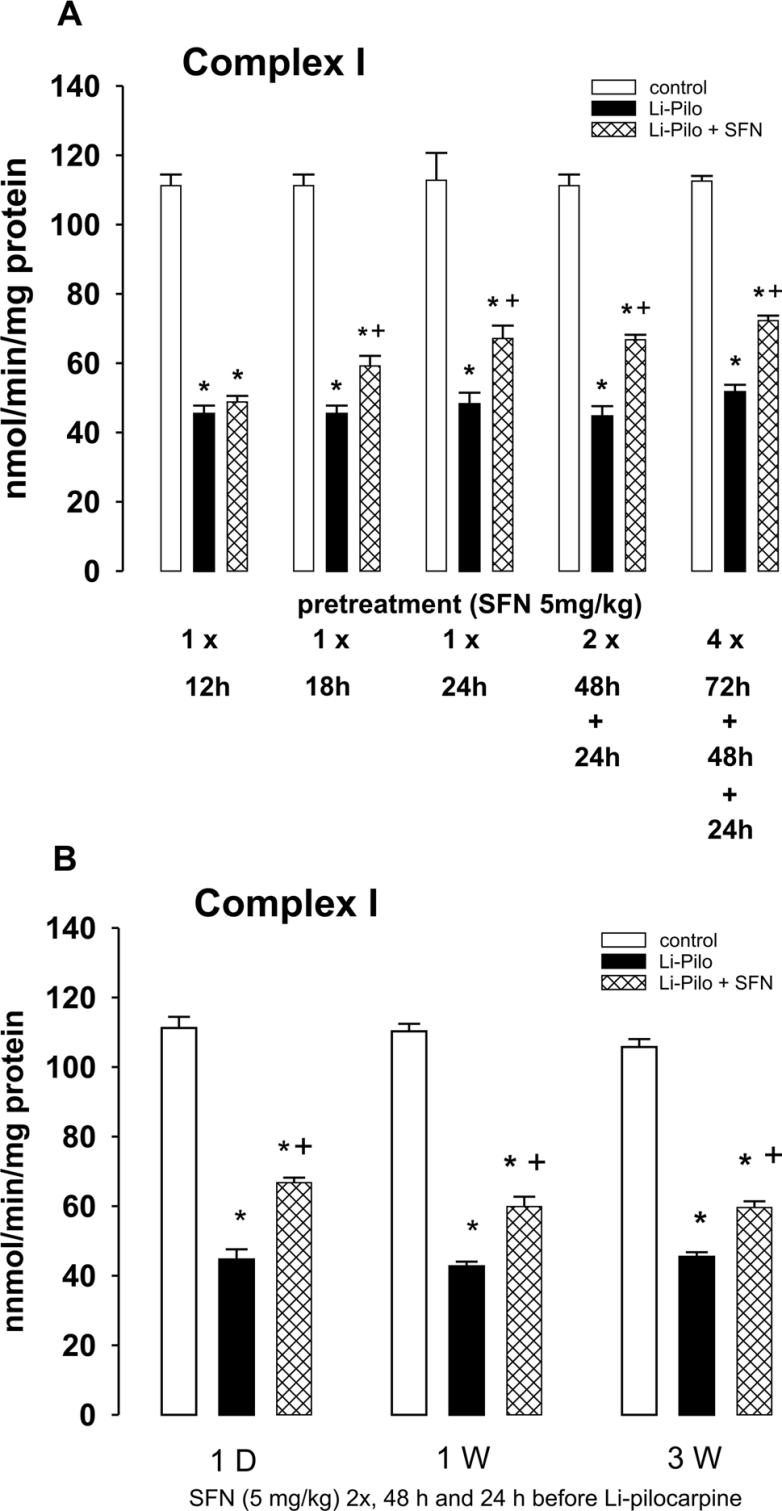
Effect of sulforaphane (SFN) on mitochondrial complex I activity. **A** Testing different doses of SFN and various time intervals of pretreatment. White columns, saline treated controls; black columns, Li-Pilo alone; cross-hatched columns, Li-Pilo plus SFN. Results are mean values for 4–6 mitochondrial preparations ± SEM. ^*^P < 0.05 as compared with appropriate controls; ^ϯ^*P* < 0.05 as compared with Li-Pilo alone. **B** Effect of SFN on mitochondrial complex I activity at 1 day and 1 and 3 weeks, respectively, following SE induced by Li-Pilo. White columns, control animals; black columns, Li-Pilo alone; cross-hatched columns, Li-Pilo plus SFN. Results are mean values for 4–6 mitochondrial preparations ± SEM. ^*^P < 0.05 as compared with appropriate controls; ^ϯ^*P* < 0.05 as compared with Li-Pilo alone

As can be seen in Fig. 3A, B, a pronounced decrease of mitochondrial complex I activity (~ 60%) occurred at all the studied periods following SE, i.e., at 1 day, 1 week, and 3 weeks, respectively. To evaluate the potential protective effect of SFN, we have decided to find out an optimal dose and time for pretreatment with SFN. Figure 3A demonstrates that already the dose of 5 mg/kg given 18 h before the onset of SE, attenuated significantly the inhibition of complex I activity. It is also evident that increasing the concentration of SFN above a certain level does not increase further the extent of protection. Based on these findings, we have selected to employ for all experiments 2 doses of SFN (5 mg/kg each), given 48 h and 24 h before the induction of SE.

As evident in Fig. 3B, this mode of treatment provided a marked attenuation of complex I deficiency at all the studied time points. Thus, approximately 40–46% decrease in mitochondrial complex I activity was observed in SFN-treated groups as compared to ~ 60% inhibition in animals treated with Li-Pilo alone. The protection was significant, but not complete, as complex I activities in rat pups pretreated with SFN remained significantly lower compared with control animals.

## Discussion

The main findings of our recent study (part I) [62] have revealed that SFN pretreatment has no anticonvulsant effect in immature rats (EEG recordings performed during the acute phase of SE), it improves cerebral blood flow (evaluated by laser Doppler flowmetry) and accelerates CBF response to electrical stimulation. Concerning glucose metabolism (measured by 18F-DGµCT/PET recordings), SFN pretreatment not only reversed hypermetabolism in the acute phase, but it also improved hypometabolism, starting 1 day after SE (subacute phase) and lasting at least up to 22 days after onset of SE (latent phase). Importantly, SFN pretreatment led to a significantly increased expression of Nrf2 and Nrf2/ARE regulated enzyme SOD1 in the immature brain (detected by western blot analyses) (see also Table 1, summarizing the main findings of [62]).

**Table 1:** Effect of SNF associated with Li-pilocarpine-induced SE in immature rats (information taken from [62])

The increased expression of Nrf2 and Nrf2/ARE regulated SOD1; increase of mitochondrial complexes IV and V; decrease of S6 phosphorylation No changes in the behavioral character of SE; no changes in the number of electro-graphic spikes during the acute phase of SE Increased total blood flow and accelerated response to the electrical stimulation; improved blood flow response to SE-like stimulation Normalization of glucose hypermetabolism 1 h after SE; improvement of glucose hypometabolism 1 day and 3 weeks after SE No effect on PCr, ATP, glucose, glycogen, and lactate concentrations in the cerebral cortex 30 min after the onset of SE	

The data of the present study confirmed our previous findings concerning the existence of oxidative stress and mitochondrial dysfunction in the immature brain during both the acute phase of status epilepticus and during long periods following SE. Importantly, novel findings clearly demonstrate that sulforaphane (SFN), a natural isothiocyanate (present in cruciferous vegetables, particularly broccoli and its young sprouts), has a marked beneficial effect on both of these processes.

The important question arises whether the protective effect of SFN could not be due to an anticonvulsant effect. However, the behavior pattern of seizures (latency, severity, frequency, duration) was not influenced in our study by SFN and the absence of the anticonvulsant effect of SFN has also been confirmed by EEG recordings (see [62] Supplementary Fig. 1). It is thus unlikely that the protective effect of SFN observed in our study is due to an anticonvulsant effect. It should be mentioned that the existing data concerning adult animals are rather controversial, reporting elevated seizure thresholds in some seizure models in adult mice (6-Hz stimulation, flurothyl model) [59], whereas the significantly decreased threshold for the onset of seizures in the PTZ-model was reported by Socala et al. [68]. It should, however, be emphasized that in the latter study very high doses (200–300 mg/kg), which produced marked toxicity, were employed. The reason for the discrepancies is not clear, but it may be due to several potential factors, such as the dose of SFN, the way of treatment, the age of the animals, or the epilepsy model employed. Nevertheless, it should be noted that the absence of an anticonvulsant effect of SFN during the acute phase observed in our study does not exclude the possibility that SFN could have a disease-modifying effect during the later epileptogenesis phase, similarly as reported in adult animals (e.g., [43, 44]). This issue should be clarified by the ongoing experiments.

Thus, the findings of the present study clearly indicate that both the acute phase of SE and the early period of epileptogenesis following SE induced in immature rats are associated with oxidative stress and mitochondrial dysfunction. The presence of oxidative stress was documented by the detection of the increased levels of superoxide anion in several brain structures, using the Het assay. It should be noted that this method, despite several limitations, can be considered a useful marker of oxidative stress [69]. It can be assumed that the enhanced and long-lasting production of ROS may lead to conditions favoring oxidative modification of sensitive targets. Significantly increased levels of 3-NT and 4-HNE (two markers of oxidative damage) which we determined in mitochondrial samples are in good accordance with this assumption. Importantly, our findings have revealed that pretreatment with sulforaphane was able to prevent or significantly reduce not only the increased production of ROS but also significantly decrease the elevation of both markers of oxidative damage.

As to the mitochondrial dysfunction, deficiency of complex I is one of the most frequent dysfunctions of the mitochondrial respiratory chain in human diseases. In relation to epilepsy, impairment of complex I was observed in human patients [70, 71] and also in several experimental models of epilepsy in adults [8, 72, 73] as well as immature animals [15, 17, 24–26]. In our studies on immature rats, we have observed a marked decrease (~ 60%) of complex I activity not only during the acute phase of status epilepticus, but this decrease persisted during long periods following SE (as demonstrated in Fig. 3B and, even up to 5 weeks, the longest period studied [15]), i.e., during the period of epileptogenesis. In our previous study, we documented that the decrease was selective for complex I and it was not associated with changes in the size of the assembled complex I or with changes in the mitochondrial content of complex I [24].

We have shown that the decrease was substantially reduced by treatment with selected free radical scavengers, namely, by the SOD mimetics MnTMPYP and Tempol, by a selective peroxynitrite scavenger and decomposition catalyst FeTPPS, by resveratrol, and, as shown in the present study, by sulforaphane. Interestingly, similar findings were found by Luis-Garcia et al. [58]. These authors, using the rodent experimental model of Huntington’s disease induced by quinolinic acid, reported that sulforaphane attenuated effectively mitochondrial dysfunction by preventing the decrease of respiratory control ratio, transmembrane potential, ability to synthesize ATP, and, importantly, deficiency of complex I activity.

Based on all these findings and the well-established extreme sensitivity of complex I to both oxidative and nitrosative stress ([24] and references therein), the main mechanism underlying the sustained complex I deficiency is likely oxidative modification localized on some critical subunit of complex I. Several possibilities can be considered, such as nitration of tyrosine and/or tryptophan residues within the complex, S-nitrosation of some of its protein thiols, oxidation of iron-sulfur clusters, and others. Murray et al. [74], e.g., reported that exposure of bovine heart mitochondria to peroxynitrite resulted in a significant inhibition of complex I and at the same time five of its 46 subunits contained 3-NT. Interestingly, tyrosine nitration has also been detected in glutamine synthetase, accompanied by the reduction of enzyme activity in the pentylenetetrazol model of epilepsy [75]. In the kainate model of SE in adult rats, Ryan et al. [76] reported an increased level of protein carbonyls concomitantly with decreased activity of complex I. Mass spectrometry analysis identified specific metal-catalyzed carbonylation to arginine 76 within the 75 kDa subunit of complex I. Computational-based molecular modeling studies predicted that carbonylation at this site can induce substantial alterations to the protein complex, leading to impaired function.

As already mentioned above, it is unlikely that the observed protective effect of SFN is due to an anticonvulsant effect. It is tempting to assume that the most likely mechanism underlying the protective effect is the ability of SFN to activate the Nrf2/ARE signaling pathway, as documented by many studies of various disorders in adult animals (for references see above). That this mechanism is also active in the immature brain has been shown in neonatal ischemic-hypoxia models [60, 61]. In addition, our recent study [62] has confirmed by WB analysis that in brains of immature 12-day-old rats SFN causes significantly increased expression of Nrf2 and Nrf2/ARE regulated enzyme SOD1.

There have been discovered two main mechanisms of Nrf2 activation, namely, canonical (covalent) and non-canonical (non-covalent) [77]. As to the canonical mechanism, Keap 1 has several reactive cysteine residues (sensors) which are by Nrf2 inducers chemically modified (by oxidation or alkylation), leading to a conformation change and a subsequent release of Nrf2 [38, 78]. However, this mechanism may be associated with unspecific activity due to potential interaction with cysteines of other important cellular proteins. A more recently discovered non-canonical Nrf2 activation pathway is based on the ability of certain compounds (peptides or small molecules) to disrupt the Keap1–Nrf2 complex by direct interaction with Keap 1 or Nrf2 [79, 80]. Recent findings suggest that Nrf2 activation through direct inhibition of the protein–protein interaction is more prolonged than that produced by classical inducers and importantly, maybe more specific, with less of “off-target” effects. The direct small molecule inhibitors of the Keap1-Nrf2–PPI thus appear to be the most promising strategy for the activation of Nrf2.

It should also be mentioned that the upregulation of specific protein kinases that phosphorylate Nrf2 facilitates its dissociation from Keap1 [81]. Protein kinase C, e.g., has been shown to phosphorylate Nrf2 Ser^40^ within the Neh2 domain of Nrf2 and caused the dissociation of Nrf2 from its repressor Keap 1, promoting thus Nrf2 transcription activity [82]. Kinase-mediated phosphorylation modifications are thus important posttranslational regulators of Nrf2 activity. It should, however, be emphasized that the phosphorylation modification of Nrf2 may affect, according to the phosphorylated sites within each structural domain of Nrf2, different processes, i.e., not only Nrf2 nuclear translocation but also Nrf2 proteasomal degradation or Nrf2 nuclear export [81].

As far as SFN is concerned, mass spectrometry-based studies of Hu et al. [83] have confirmed that SFN reacts with at least four cysteine residues of Keap1, including C151. Interestingly, C151 was established also as the primary sensor for RTA 408 (see [44]). In addition, Zhao et al. [84], using a cellular model of AD, reported an epigenetic modification of Nrf2 by sulforaphane and showed that SFN upregulated the expression of Nrf2 and promoted its nuclear translocation through decreasing DNA methylation levels of the Nrf2 promoter.

As already mentioned, current therapies for patients with epilepsy have been mostly symptomatic and not fully effective. Identifying new potential targets for more efficient therapies is thus urgently needed. These targets should be selected on the basis of the revealed key mechanisms underlying the disease. Recent studies of many laboratories and also our group have documented that enhanced ROS and/or RNS production, induction of oxidative stress and damage, and mitochondrial dysfunction play a crucial role in the pathophysiology of status epilepticus. It seems thus reasonable to predict that the treatment with antioxidants as an “add-on” therapy should have a beneficial effect [85]. In addition, this supposition is supported by the fact that the existing antiseizure drugs were not able to influence the increased levels of oxidative markers in patients with epilepsy [86]. Findings of many studies suggest that preferable to exogenous antioxidants, substances possessing the ability to increase the endogenous antioxidant defenses of cells (or potentially a combination of both of these approaches [50]) may provide a better and long-lasting effect. In this connection, compounds that can activate Nrf2/ARE signaling pathway seem to be an ideal candidates.

## Conclusion

The present findings indicate that both the oxidative stress and mitochondrial dysfunction associated with SE induced in immature rats by Li-pilocarpine can be prevented or significantly reduced by pretreatment with sulforaphane, a natural isothiocyanate and well-known activator of Nrf2. Since sulforaphane had no direct anti-seizure effect, the findings suggest that the ability of sulforaphane to activate Nrf2 is most likely responsible for the observed protective effect. Nrf2/ARE signaling pathway can be considered a promising target for novel therapies of several disorders inclusive epilepsy, particularly when new compounds, possessing inhibitory activity against protein–protein interaction (PPI) between Nrf2 and its repressor protein Keap 1, with less of “off-target” effects and, importantly, with an optimal permeability and bioavailability properties, become available commercially.

## Data Availability

The datasets generated and analyzed during the current study and supporting the results reported in this article are available in the repository of J. Folbergrová and J. Otáhal, Institute of Physiology, Czech Academy of Sciences, Prague.
